# Bis-cyclopropane analog of disorazole C_1_ is a microtubule-destabilizing agent active in abcb1-overexpressing human colon cancer cells

**DOI:** 10.18632/oncotarget.5885

**Published:** 2015-10-19

**Authors:** Shaoyu Wu, Zhijian Guo, Chad D. Hopkins, Ning Wei, Edward Chu, Peter Wipf, John C. Schmitz

**Affiliations:** ^1^ Division of Hematology-Oncology, Department of Medicine, University of Pittsburgh School of Medicine, Pittsburgh, PA 15232, USA; ^2^ Cancer Therapeutics Program, University of Pittsburgh Cancer Institute, University of Pittsburgh, Pittsburgh, PA 15213, USA; ^3^ Department of Chemistry, School of Pharmaceutical Science, Southern Medical University, Guangzhou 510515, China; ^4^ Department of Nephrology, NanFang Hospital, Southern Medical University, Guangzhou 510515, China; ^5^ Department of Chemistry, University of Pittsburgh, Pittsburgh, PA 15260, USA

**Keywords:** tubulin polymerization, disorazole, human colorectal cancer

## Abstract

The novel, chemically stabilized disorazole analog, (−)-CP_2_-disorazole C_1_ (**1**) displayed potent anti-proliferative activity against a broad-spectrum of human colorectal cancer cells. HCT15 and H630R1 cell lines expressing high basal levels of the ABCB1 protein, known to cause multi-drug resistance, were also sensitive to growth inhibition by **1** but were resistant to both vincristine and docetaxel, two commonly used microtubule inhibitors. Compound **1** exhibited strong inhibition of tubulin polymerization at a level comparable to vincristine. In addition, treatment with **1** resulted in decreased protein levels of β-tubulin but not α-tubulin. An analysis of cellular proteins known to interact with microtubules showed that **1** caused decreased expression of c-Myc, APC, Rb, and additional key cellular signaling pathways in CRC cells. Treatment with compound **1** also resulted in G2/M cell cycle arrest and induction of apoptosis, but not senescence. Furthermore, endothelial spheroid sprouting assays demonstrated that **1** suppressed angiogenesis and can, therefore, potentially prevent cancer cells from spreading and metastasizing. Taken together, these findings suggest that the microtubule disruptor **1** may be a potential drug candidate for the treatment of mCRC.

## INTRODUCTION

Approximately 30% of all anticancer drugs used globally are derived from plant and/or animal sources. In spite of a recent focus in the pharmaceutical industry on biological agents as well as chemical lead structures identified in screens of large heterocyclic compound libraries, natural products continue to play a significant role as a potential source of antitumor agents [1–4]. Since the isolation of novel lead compounds has become more time- and resource-intensive, a critical need to develop alternative strategies to traditional prospecting on land and in the sea is emerging. In addition to biosynthetic approaches and the manipulation of gene clusters [5, 6], the synthesis of novel natural product-like compounds offers interesting new opportunities to augment traditional natural product extract collections [7–9]. An alternative discovery strategy involves the structural modification of natural products with the goals of enhancing physico-chemical properties and target selectivity, while reducing complexity and facilitating scale-up. However, this approach is often limited by narrow structure-activity relationships and significant reductions in potency for even slightly modified structural variants [10–12].

**Figure 1:**
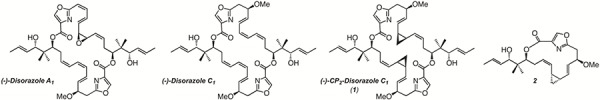
Structures of natural disorazoles and synthetic analogs

The disorazoles comprise a group of approximately 30 structurally closely related polyene macrodiolides, first isolated from the mycobacterium *Sorangium cellulosum* in 1994 [13–15]. A major fermentation product, disorazole A_1_, blocked cancer cell proliferation at picomolar concentrations and inhibited *in vitro* polymerization of tubulin. Since the highly electrophilic divinyl oxirane moiety of A_1_ is not considered to be a pharmacologically desirable drug-like moiety, we selected a minor fermentation component, disorazole C_1_, which lacked the reactive epoxide component, as a higher priority target for chemical synthesis [16]. Subsequent biological studies indicated that the vinyl oxirane moiety was not critical for antiproliferative activity as disorazole C_1_ maintained low nanomolar anticancer properties, which were also correlated with microtubule destabilization [17–20]. Furthermore, our group recently succeeded in the synthesis of a bis-cyclopropyl analog of disorazole C_1_, (−)-CP_2_-disorazole C_1_ (**1**), that retained low-nanomolar biological activity similar to what was observed with the parent compound (Figure 1) [21]. We speculated that replacement of the central (*Z*)-alkene in the (*Z,Z,E*)-triene subunit of disorazole C_1_ with a cyclopropane moiety would generate an even more chemically stable analog with minimal perturbation of the conformation of the macrocycle that was still capable of engaging the biological target [22, 23]. Herein, we conducted a series of experiments to further evaluate the biological effects and potential mechanism of action of (−)-CP_2_-disorazole C_1_ (**1**) in human colon cancer cells.

## RESULTS

### Anti-proliferative activity of (−)-CP_2_-disorazole C_1_ (1) against a broad spectrum of human colorectal cancer cells

We previously showed that **1** had significant anti-proliferative activity against human colon cancer RKO, HCT116, and H630 cells. In contrast, the cyclic monomer **2** had no inhibitory effect on cell growth at concentrations up to 1,000-fold higher [21]. In the present study, we further investigated the growth inhibitory effect of **1** in various human CRC cell lines with different genetic backgrounds. As shown in Table 1, IC_50_ values for **1** ranged from 4 to 26 nM. Growth inhibition was similar in both p53+/+ and p53−/− HCT116 cells, suggesting that the cytotoxic effect of this disorazole analog was mediated through p53-independent pathways. This phenomenon has been demonstrated previously for agents that destabilize microtubules [24, 25]. HCT116 p21−/− cells, which are p21 deleted, were slightly more sensitive to growth inhibition with **1**, but this effect was not statistically significant. Interestingly, HCT15 and H630R1 cells were both sensitive to growth inhibition by **1**, but were significantly more resistant to vincristine and docetaxel, two anti-microtubule inhibitor agents that are commonly used in clinical practice to treat a broad range of human cancers. An immunoblot analysis revealed that these two cell lines expressed relatively high basal levels of the ABCB1 protein (Supplementary Figure S1), which is known to mediate multi-drug resistance. In contrast, RKO and HCT116 cells had undetectable levels of ABCB1. Increased expression of ABCB1 has been identified as a resistance mechanism to disorazoles C_1_ and A_1_ as well as to the vinca alkaloids and paclitaxel [17, 26–28]. This result suggests that the structural modifications to **1** may have overcome this potential resistance mechanism. We observed no detectable levels of ABCG2 protein in the human CRC cell lines. Other ABC-related genes such as ABCC1 were not investigated given that cells remained sensitive to **1**. Of note, we were unable to determine an IC_50_ value for **1** in normal colon epithelial 841 and FHC cells suggesting that this analog does not have cytotoxic effects against normal cells and that it displays an expanded therapeutic window. However, these normal cells were also resistant to vincristine- and docetaxel-mediated growth inhibition. To determine whether **1** could inhibit the ability of CRC cells to repopulate from single cells, we tested the effects of **1** on colony formation. As shown in Table 2, **1** effectively inhibited clonogenic growth in all the CRC cell lines tested. The ABCB1-overexpressing cells lines, while still sensitive to **1**, had IC_50_ values significantly higher than the ABCB1-negative cell lines.

**Table 1:** Effect of microtubule inhibitors on human colon cancer cell proliferation

Cell line	IC_50_ (nM)		
	1	Vincristine	Docetaxel
RKO	9.63 ± 4.30	1.53 ± 0.66	0.78 ± 0.03
HCT116	7.94 ± 1.86	3.76 ± 0.60	0.64 ± 0.17
HCT116 p53^−/−^	6.10 ± 1.50	2.95 ± 0.29	2.39 ± 0.60
HCT116 p21^−/−^	3.82 ± 1.69	3.95 ± 0.86	2.94 ± 0.78
HCT15	14.06 ± 1.93	48.25 ± 4.04	55.43 ± 8.84
H630	19.03 ± 4.74	8.76 ± 0.76	1.26 ± 0.13
H630 R1	26.12 ± 3.97	221.08 ± 55.78	112.66 ± 32.34
841	> 300	172.30 ± 37.24	171.30 ± 20.48
FHC	> 300	94.18 ± 16.28	119.85 ± 31.20

IC_50_ values denote the drug concentration that inhibits 50% of cell growth. Values represent the mean ± S.D. from 3–5 separate experiments.

**Table 2:** Effect of 1 on clonogenic growth

Cell line	IC_50_ (nM)
RKO	1.89 ± 0.06
HCT116	0.84 ± 0.37
HCT116 p53^−/−^	1.40 ± 0.13
HCT116 p21^−/−^	1.35 ± 0.15
HCT15	9.13 ± 0.51
H630	7.76 ± 1.09
H630 R1	8.24 ± 1.82

IC_50_ values represent the mean ± S.D. from 3–5 separate experiments.

### Compound 1 inhibited tubulin polymerization

**Figure 2:**
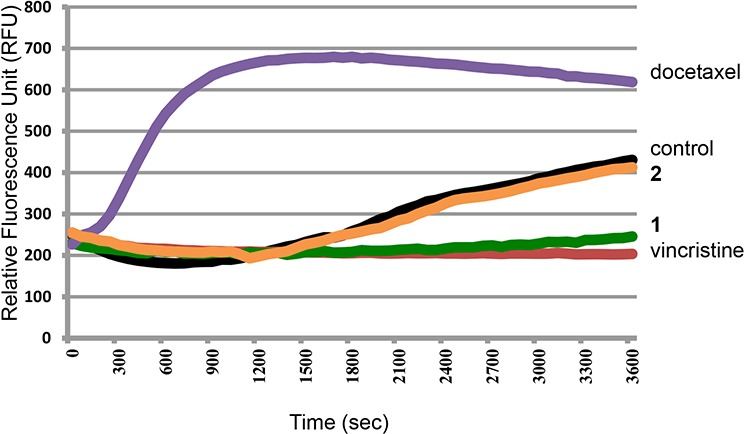
Effect of microtubule inhibitors on tubulin polymerization Recombinant tubulin protein was incubated alone (control) or with 3 μM docetaxel, **2**, **1**, or vincristine. Each condition was tested in duplicate. Tubulin polymerization was measured by excitation at 360 nm and emission at 450 nm.

To investigate the direct interaction between tubulin and the disorazole analog **1**, a cell-free tubulin polymerization assay was used. Tubulin polymerization was monitored by the incorporation of a fluorescent marker into microtubules. As recombinant tubulin protein polymerized, it showed a pattern of nucleation, growth, and eventual steady-state equilibrium (Figure 2). In the presence of docetaxel, a microtubule stabilizing molecule [1], the nucleation period was vastly decreased, the growth rate faster, and the final polymer mass was greater. In the case of the microtubule disrupting agent, vincristine, there was a decrease in the growth rate and a significant reduction in the final polymer mass. Compound **1** exhibited inhibition of microtubule formation in a manner comparable to vincristine, suggesting that **1** possesses strong anti-tubulin polymerization activity. In contrast, the inactive disorazole analog **2** had absolutely no effect on tubulin polymerization.

**Figure 3:**
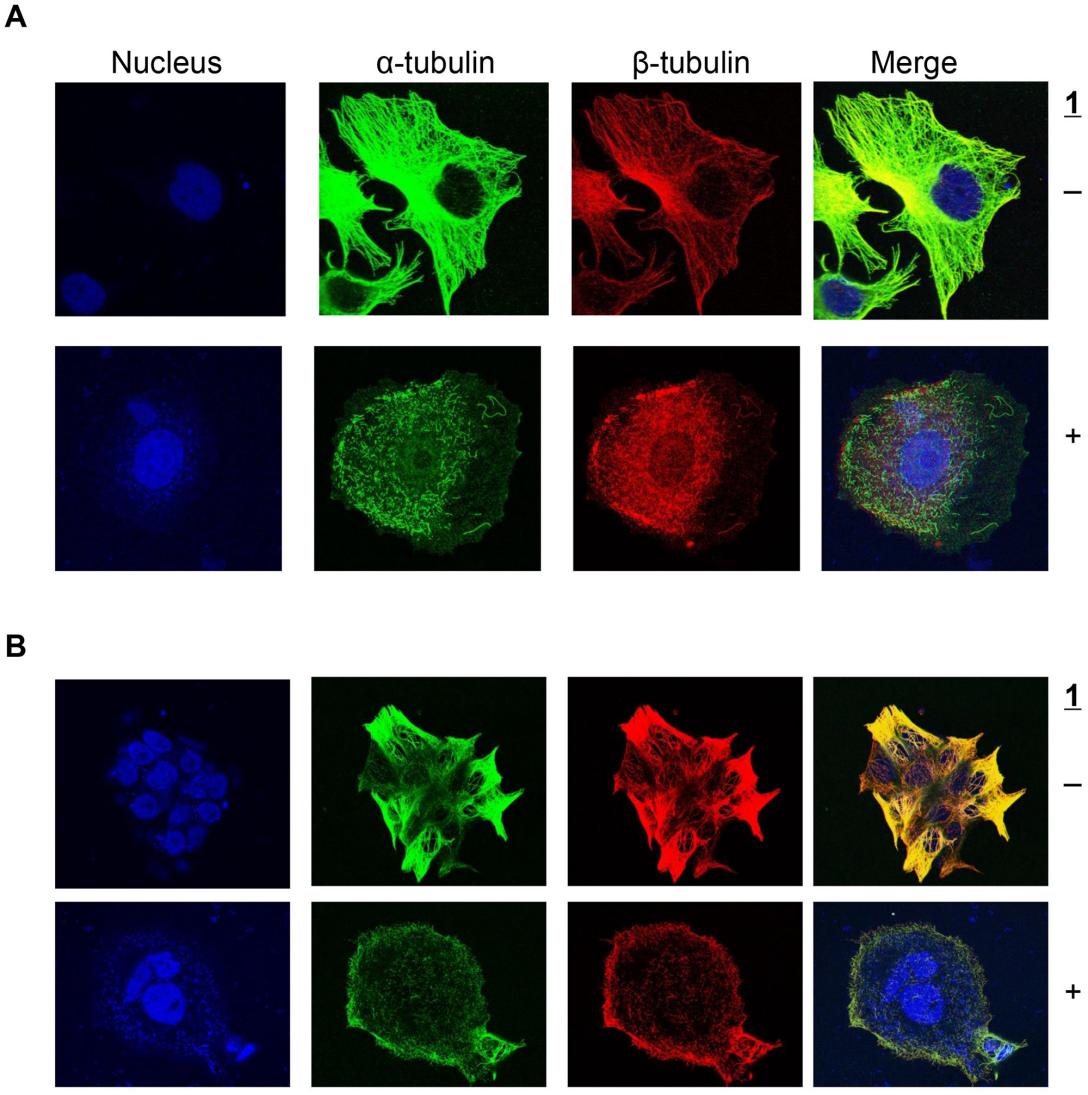
Effect of 1 on α-tubulin and β-tubulin expresion H630 **A.** and H630R1 **B.** cells were seeded on glass coverslips, incubated with **1** (20 nM) for 18 h, then fixed and processed for confocal microscopy as described in the Materials and Methods section.

We next investigated the effect of **1** on tubulin organization in intact cells and the microtubule structure of cells was visualized via immunocytochemistry. As shown in Figure 3, both α-tubulin and β-tubulin microtubules formed an intact network of tubules extending into the lamellipodium in untreated cells. Treatment of H630 and H630R1 cells with **1** resulted in significant disruption of both tubulin subtypes, loss of cellular structure, and formation of cell membrane rounding. Following drug treatment, both α-tubulin and β-tubulin appeared as a punctate dot-staining pattern.

### Effect of compound 1 on expression of tubulin and its subtypes

**Figure 4:**
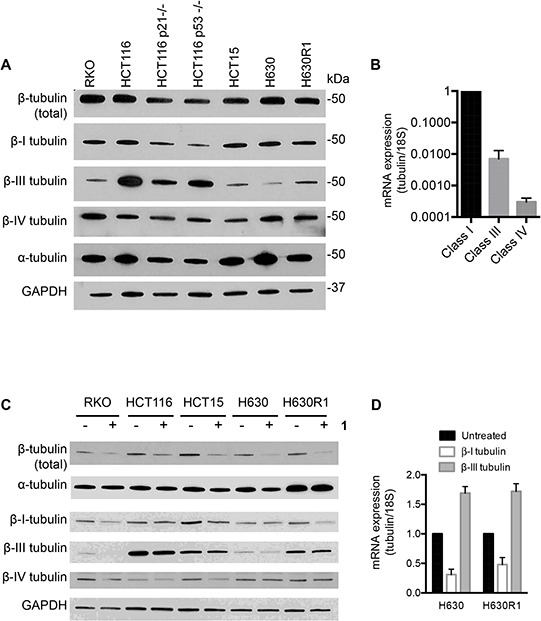
Basal tubulin protein expression in human colorectal cancer cell lines **A.** Immunoblot analysis of β-tubulin isotypes. **B.** Basal mRNA expression of β-tubulin isotypes in H630 cells. **C.** Effect of **1** on tubulin protein and mRNA expression. Cells were treated with or without **1** (20 nM) for 24 h and then processed for immunoblot analysis or qRT-PCR **D.** A representative immunoblot is shown from four independent experiments. (D) mRNA values represent the mean ± S.D. from four experiments.

**Figure 5:**
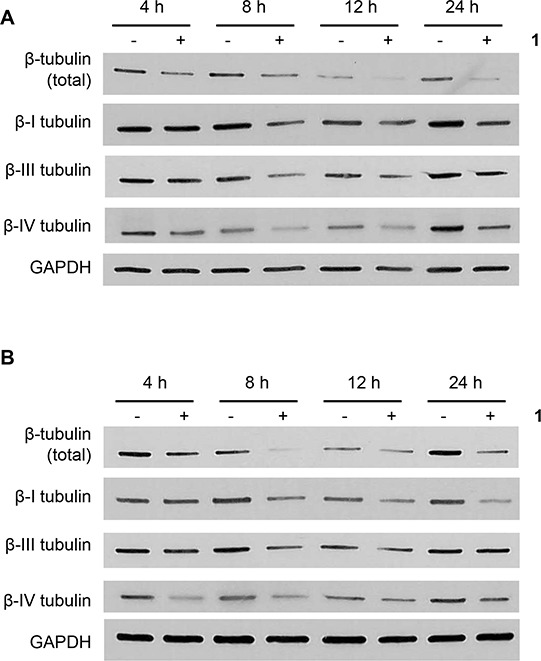
Time-dependent effects of 1 on β-tubulin expresion H630 **A.** and H630R1 **B.** cells were plated and treated with **1** (20 nM). At various times, cells were harvested and processed for immunoblot analysis. A representative blot from four independent experiments is shown.

**Figure 6:**
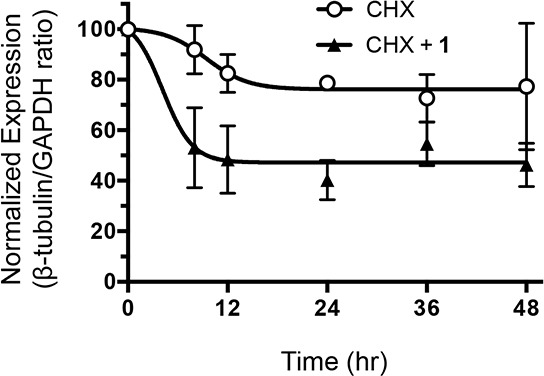
Effect of 1 on β-tubulin protein stability H630 cells were incubated with cycloheximide (10 μM) in the presence or absence of **1** (20 nM). At various times, cells were harvested and β-tubulin protein levels were assessed by immunoblot analysis. Protein expression was quantified by ImageJ and normalized to GAPDH expression.

Microtubules are heterodimers composed of combinations of eight α- and seven β-tubulin isotypes [2, 29]. Class I and IV have been identified as the major β-tubulin isotypes present in colon tumors and colon cancer cell lines with minor expression of class III and V [30, 31]. Using isotype-specific antibodies, we were able to detect class I, III, and IV β-tubulin proteins in human CRC cells (Figure 4A). These isotypes were expressed at similar levels in all cell lines except for class III, which was highly expressed in HCT116 cells. At the mRNA level, the class I isotype comprised the majority of β-tubulin mRNA with class III and class IV expressed at 100-fold and 1,000-fold lower levels, respectively (Figure 4B). We then evaluated the expression level of these isotypes after treatment with **1**. Exposure of CRC cell lines to **1** for 24 h resulted in decreased levels of total β-tubulin protein but not α-tubulin (Figure 4C). The inactive cyclic monomer **2**, used as a control compound to highlight the specificity of **1**, had no effect on β-tubulin expression (Supplementary Figure S2). Treatment with **1** resulted in decreased expression of β-I tubulin protein in RKO, HCT15, and H630R1 cells. Isotype β-III tubulin was decreased only in RKO cells, while β-IV tubulin was decreased in RKO and HCT15 cells. Exposure to **1** had a differential effect on tubulin mRNA expression as β-I tubulin mRNA decreased significantly while there was a paradoxical increase in β-III tubulin mRNA expression (Figure 4D). We next determined the time-dependent effect of **1** on β-tubulin expression. After 4 h, total β-tubulin expression decreased significantly in H630 cells but not in H630R1 cells (Figures 5A, 5B). However, by 8 h, tubulin expression was similarly suppressed in H630R1 cells. Levels of β-I tubulin and β-IV tubulin decreased rapidly and remained suppressed for 24 h. Of note, expression of β-III tubulin decreased initially but returned to baseline levels by 24 h. This finding coincides with the observed induction of β-III tubulin mRNA (Figure 4D). To investigate the mechanism by which tubulin levels were decreased, expression of tubulin was determined in the presence of the protein synthesis inhibitor cycloheximide. As shown in Figure 6, treatment with **1** resulted in a rapid decrease in β-tubulin expression. However, after 12 h, no further decrease was observed suggesting that compound **1** did not alter the stability of tubulin after the initial loss of protein.

### Effect of compound 1 on migration

**Figure 7:**
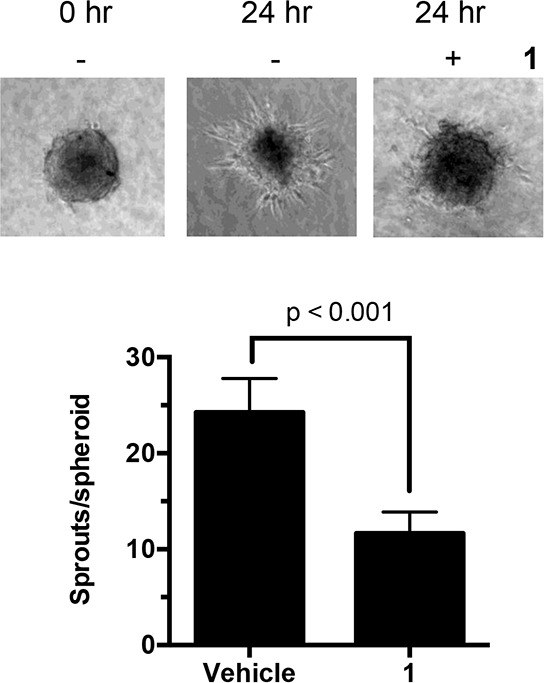
Effect of 1 on endothelial sprouts Human endothelial cell spheroids embedded in collagen were incubated with **1** (2 nM). After 24 h, the number of sprouts in each spheroid was counted manually. Values represent the mean ± S.D. from 15–17 individual spheroids.

In addition to regulating cell proliferation, microtubules play a key role in cell migration. With this in mind, *in vitro* endothelial spheroid sprouting assays were performed to test the effect of **1** on the angiogenesis of the microvascular endothelial cells. After spheroid formation and embedding into collagen gel, the spheroids were treated with compound **1** for 24 h. In the untreated spheroids, sprouts grew out around the spheroid at the frequency of 24 sprouts/spheroid (Figure 7). Treatment with **1** significantly reduced the number of sprouts by 50% to 12 sprout/spheroids. This finding suggests that this disorazole C_1_ analog can potentially suppress the ability of cells to invade and metastasize.

### Effects of compound 1 on microtubule-related proteins

**Figure 8:**
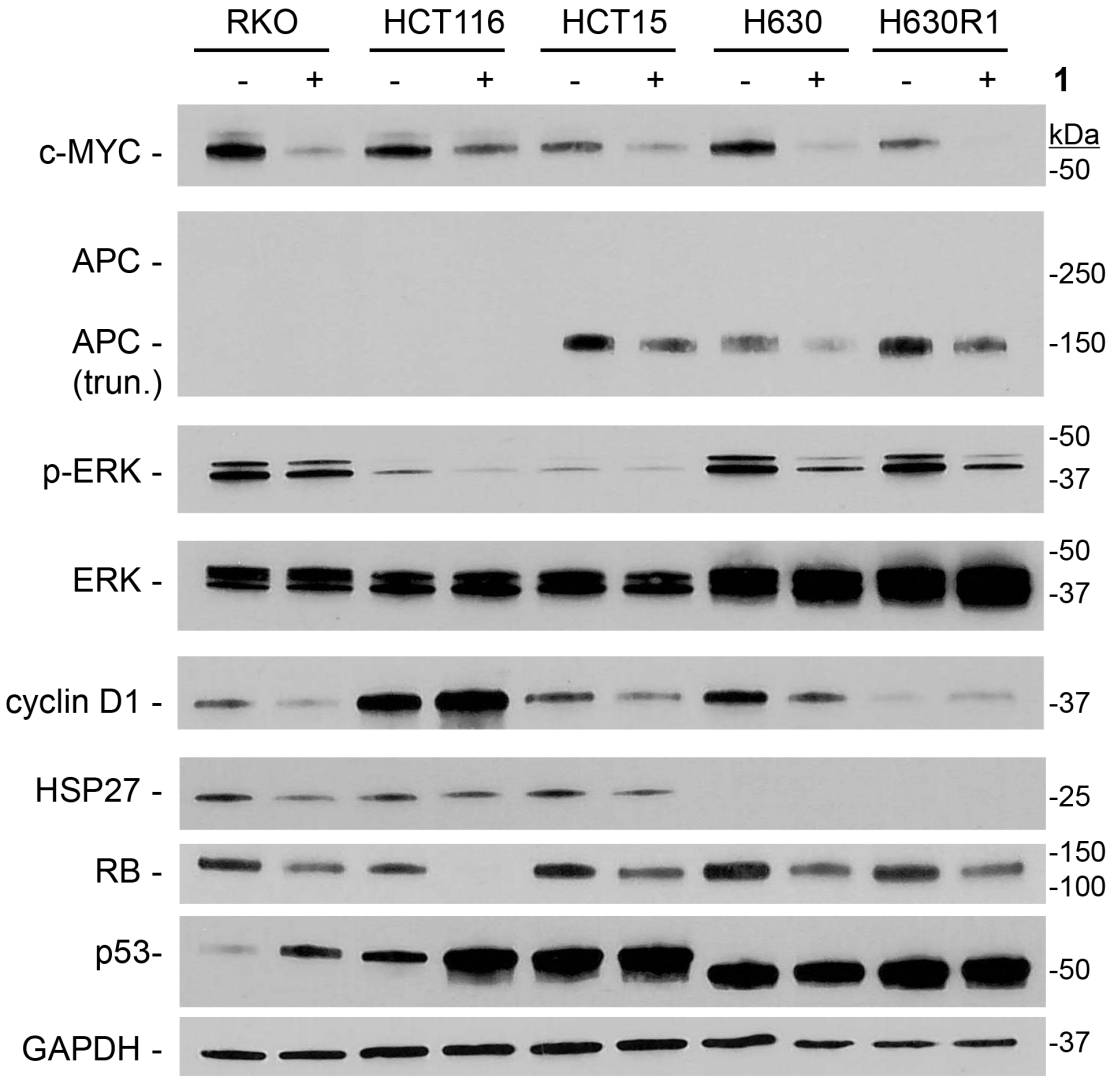
Effect of 1 on expression of microtubule-interacting proteins Cells were treated with or without **1** (IC_50_ values) for 24 h, and processed for immunoblot analysis. Representative blots from four experiments are shown.

A large number of cellular proteins are known to associate with microtubules [29, 32]. We next determined whether the decrease in β-tubulin expression resulting from treatment with **1** was associated with altered expression of these microtubule-associated proteins. The c-Myc transcription factor has been shown to interact and bind α-tubulin [33]. Exposure to compound **1** resulted in significant reduction in c-Myc protein expression in all cell lines (Figure 8). The Adenomatous Polyposis Coli (APC) tumor suppressor is also known to bind microtubules and β-catenin. Treatment with **1** resulted in decreased expression of the truncated mutant form of APC in HCT15, H630, and H630R1 cells. We were unable to detect full-length APC in RKO and HCT116 cells. We observed significant reduction in p-ERK signaling in all cell lines with total ERK remaining unchanged. The effects of **1** on other microtubule-interacting proteins were cell-line specific. Expression of cyclin D1 decreased in RKO, HCT15, and H630 cells, while it was increased in HCT116 and H630R1 cells. We observed that expression of the heat shock protein HSP27 decreased in RKO and HCT15 cells, remained unchanged in HCT116 cells, and was undetectable in H630 and H630R1 cells. The retinoblastoma tumor suppressor (Rb), like c-Myc, was decreased in all cells. The tumor suppressor p53 was upregulated in cell lines containing wild-type protein (RKO; HCT116) but not in cell lines containing mutant p53 (HCT15; H630). Of note, another protein known to interact with microtubules, HSP90, was unchanged in all the CRC cell lines after treatment with **1** (data not shown).

### Effect of compound 1 on cell cycle and apoptosis

**Figure 9:**
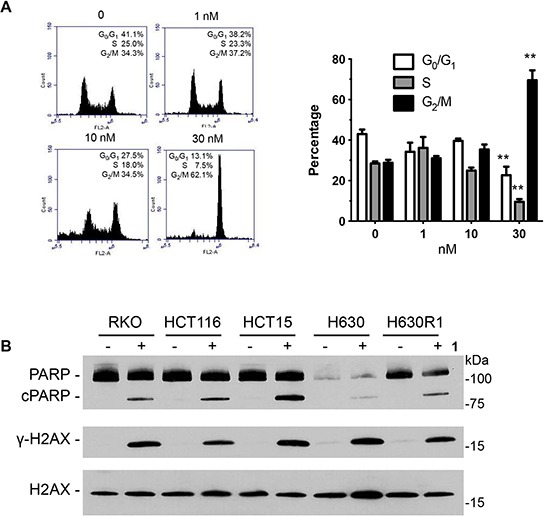
Effect of 1 on cell cycle distribution and apoptosis **A.** HCT116 cells were incubated with **1** for 24 h followed by fixation, PI staining, and analysis by flow cytometry. Values represent the mean ± S.D. from three independent experiments. **, *p* < 0.001. **B.** Cells were treated with **1** (IC_50_ values) for 24 h and processed for immunoblot analysis.

Previous studies had shown that disorazole C_1_ induced senescence [18]. Accordingly, we performed a series of cell senescence assays to investigate the potential mechanisms by which analog **1** suppressed cell growth. In contrast to the parent compound, we were unable to detect positive β-galactosidase staining in RKO and HCT116 cells upon exposure to **1** (data not shown). We then determined whether the modified disorazole might alter cell cycle distribution by performing flow cytometry analysis. Concentrations slightly higher than the IC_50_ value (30 nM) arrested cells in G_2_/M phase after 24 h (Figure 9A). The parent disorazole C_1_ had similar effects on cell cycle distribution [18]. After 48 h, cells accumulated in the sub-G_0_ phase of the cell cycle. Given this observation, we measured the apoptotic cell population with Annexin V/PI staining by flow cytometry. Table 3 confirms that **1** induced apoptosis after 48 h. No significant apoptosis was observed after 24 h. Cleavage of PARP, which is well-established as a marker of apoptosis, was observed in all cell lines after treatment with **1** at their respective IC_50_ concentrations (Figure 9B). The DNA damage marker γ-H2AX was also measured by immunoblot analysis, and it was found to be significantly induced in the CRC cell lines treated with compound **1**.

**Table 3:** Effect of 1 on apoptosis in HCT116 cells

Concentration (nM)	Viable	Early apoptosis	Late apoptosis	Necrosis
0	94.8 ± 1.6	2.7 ± 1.0	1.9 ± 0.7	1.9 ± 0.6
1	92.8 ± 0.3	3.1 ± 0.9	3.1 ± 0.1	1.0 ± 0.1
10	70.3 ± 4.7	13.9 ± 6.0	11.1 ± 1.6	4.7 ± 0.2
30	60.4 ± 6.5	26.2 ± 7.5	12.8 ± 1.5	1.8 ± 0.7

HCT116 cells were treated with 1 for 48 h prior to staining with PI and Annexin V and analyzed by flow cytometry. Values represent the mean ± S.D. from three independent experiments.

## DISCUSSION

Inhibitors of tubulin polymerization based on natural products have played a key role in cancer treatment since the anti-microtubule inhibitor vincristine, isolated from the Madagascar periwinkle plant, was FDA-approved in 1963 [34, 35]. The mechanisms of resistance to this class of compounds include overexpression of the drug efflux pump protein ABCB1, microtubule cytoskeletal changes, and overexpression of specific β-tubulin isotype and microtubule-associated proteins [36]. To overcome these resistance mechanisms, research has remained active in this field as new analogs and derivatives are continuously being developed. In the present study, we characterized the growth inhibitory effects and the biological mechanisms of action of (−)-CP_2_-disorazole C_1_ (**1**), a bis-cyclopropane stabilized analog of the antimitotic natural product disorazole C_1_ [21], against human colorectal cancer cells (CRC). Our results demonstrate that **1** inhibited cell proliferation of a wide range of human CRC cells including ABCB1-overexpressing cells resistant to both vincristine and paclitaxel. The microtubule-stabilizing agents, such as epothilones, have demonstrated similar activity in ABCB1-overexpressing cells [26, 37]. Treatment of cells with **1** also led to a disruption in microtubule formation and blocked tubulin polymerization *in vitro*, suppressed protein expression of β-tubulin subtypes in a time-dependent manner, altered expression of microtubule-related proteins, induced apoptosis, and led to G_2_/M cell cycle blockade. Taken together, these findings suggest that the novel microtubule disruptor **1** may be a potential drug candidate for treatment of mCRC.

While the highly electrophilic disorazole A_1_ is not favorable from a therapeutic perspective, a minor component of the isolate, disorazole C_1_, which does not contain the reactive vinyl oxirane moiety, was chemically synthesized [16] and subsequently found to also disrupt microtubule integrity and inhibit cell proliferation, albeit at higher concentrations compared to A_1_. Because the triene subunit of C_1_ is labile and biologically vulnerable, isosteric bis-cyclopropanes were designed within the triene structure as they also mimic the structure of the oxirane in disorazole A_1_. Preliminary data with (−)-CP_2_-disorazole C_1_ (**1**) revealed that that this molecule maintained cellular cytotoxicity against a series of human CRC cell lines [21]. Herein, we demonstrate that **1** is similar to its parent disorazole C_1_ in that it disrupts microtubule formation, displays potent nanomolar cytotoxicity against a panel of human CRC cell lines, and blocks the cell cycle in the G2/M phase. However, we did not observe senescence as had been detected previously after C_1_ treatment. Interestingly, analog **1** caused DNA damage as measured through induction of γ-H2AX whereas the parent disorazole C_1_ did not. Thus, it is conceivable that a structural change related to the bis-cyclopropane insertion may have altered the binding mode of the modified disorazole to β-tubulin resulting in subsequent changes on downstream signaling pathways.

Disorazoles are thought to bind β-tubulin uniquely among known microtubule destabilizers such as vinblastine and dolastatin [18]. While all tested family members have demonstrated the ability to disrupt microtubule formation, we show that treatment with **1** also resulted in the loss of all three β-tubulin isotypes identified in human CRC cells with no effect on α-tubulin expression. Interestingly, expression of β-III tubulin returned to baseline levels after 24 h, which also coincided with an increase in the respective mRNA levels. Overexpression of β-III tubulin in various solid tumors may predict poor patient outcome suggesting that upregulation of β-III tubulin mRNA may represent a potential resistance mechanism to TBAs. In addition to altering tubulin expression, we observed significant effects on proteins known to associate with microtubules. Previous studies have shown c-Myc to bind α-tubulin and to co-localize in the cytoplasm [33]. Bourgarel-Rey et. al. demonstrated that supra-high levels of vinblastine (10 μM) can reduce c-Myc mRNA levels in HT29-D4 cells [38]. In sharp contrast, compound **1**, at nanomolar concentrations, potently suppressed c-Myc protein expression in all human CRC cell lines. Similarly, APC was decreased in these cells following treatment with **1**. APC, known for its role in regulating the Wnt signaling pathway, is an RNA binding protein that binds to the 3′-UTR of several tubulin subtypes directing mRNA localization and synthesis in the vicinity of microtubule ends [39]. In a similar manner, the tumor suppressor p53 protein also binds and travels along tubulin. Previous studies have shown that TBAs disrupt p53 nuclear accumulation [40]. However, treatment with **1** resulted in an increased expression of p53 in p53 wild-type cell lines. Disorazole C_1_ was also able to increase p53 expression, which was attributed to induction of senescence. Other microtubule-associated proteins such as pERK, HSP27, cyclin D1, and Rb, were similarly decreased after treatment with **1**. These alterations in protein expression confirm recent studies demonstrating that TBAs may function by inhibition of trafficking of DNA repair proteins to the nucleus [41]. However, further studies are needed to clarify the subset of cellular proteins that are directly altered by loss of β-tubulin and those that are down-regulated as a result of apoptotic processes.

In summary, our studies have shown that the bis-cyclopropane disorazole **1** is highly effective at suppressing proliferation in human colon cancer cells through disruption of microtubules. Cell lines overexpressing ABCB1 retain complete sensitivity to compound **1**. The resistance of normal colon epithelial cells to the cytotoxic effects of **1** suggests that this class of compounds may have an expanded therapeutic window. The ability of **1** to reduce endothelial spreading further suggests that this compound may also prevent spreading and metastasis of primary tumors. Future studies will focus on simplifying the chemical synthesis to permit a scale-up of **1** for evaluation in *in vivo* animal tumor models to determine the antitumor activity and toxicity profile of this class of molecules.

## MATERIALS AND METHODS

### Chemicals

(−)-CP_2_-disorazole C_1_ (**1**) and **2** were synthesized as previously described (Figure 1) [21]. Vincristine sulfate and docetaxel were obtained from Sigma-Aldrich Co. (St. Louis, MO). For *in vitro* use, compounds were resuspended in dimethyl sulfoxide (DMSO; Sigma) and the DMSO concentration never exceeded 0.1% in any experiment.

### Cell culture

The human colon cancer RKO and HCT15 cells and normal human colon epithelial CCD841 CoN and FHC cells were obtained from American Type Culture Collection (ATCC). The parental HCT116 and subclone HCT116 p53−/− and HCT116 p21−/− cell lines were kindly provided by Dr. B. Vogelstein. H630 and H630R1 cell lines were originally obtained from Dr. Adi Gazdar [42] and maintained in our laboratory. All cells (except FHC cells) were maintained in RPMI-1640 medium (Invitrogen; Carlsbad, CA) with 10% (V/V) fetal bovine serum at 37°C in a humidified incubator with 5% CO_2_. FHC cells were maintained according to ATCC guidelines. Human microvascular endothelial cell line (HMEC-1) were cultured in MCDB-131 (Life Technologies) growth medium supplemented with 10% (v/v) FBS (Corning), 1 μg/mL hydrocortisone (Sigma), 10 mM L-Glutamine (Life Technologies) and 10 ng/mL EGF (Life Technologies). Cells were tested monthly for mycoplasma by the MycoAlert Mycoplasma detection assay (Cambrex BioScience; Rockland, ME).

### Cell proliferation assay

Cells were plated in 96-well plates at a density of 1000 cells/well. On the following day, cells were incubated with **1**, vincristine, and docetaxel for at least three cell doublings (72 to 120 h). Normal epithelial cells were incubated with these same agents for 7 days. Cell proliferation was quantified by the WST-1 assay (Roche; Indianapolis, IN).

### Clonogenic assay

Cells were plated in 6-well plates at a density of 400 cells/well. On the following day, cells were treated with **1** for 24 h, after which time, the growth medium was replaced. After 8 days, cell colonies were fixed with trypan blue solution (75% methanol/25% acetic acid/0.25% trypan blue), washed, and air-dried before counting colonies >50 cells.

### Tubulin polymerization assay

Tubulin polymerization assays were conducted using the Tubulin Polymerization Assay Fluorescence kit (cat#BK011P, Cytoskeleton Inc.; Denver, CO) according to the manufacturer's instructions. Briefly, 100 μg of the reconstituted tubulin protein was added to each well of a pre-warmed 96-well plate and exposed to vehicle (0.1% DMSO), vincristine, docetaxel, **1**, or **2**. The absorbance excitation 360 nm and emission 450 nm was recorded every min for 1 h using a TECAN Safire II microplate reader (TECAN U.S. Inc., Research Triangle Park, NC) at 37°C. The dose-response curves were plotted using Prism 3 (Graphpad Software Inc.; San Diego, CA).

### Immunofluorescence microtubule detection

H630 and H630R1 cells (2 × 10^4^ cells/well) were plated on glass coverslips in 12-well plates and allowed to attach overnight. After treatment with **1** or 0.1% DMSO for 18 h, cells were rinsed twice, fixed with 2% paraformaldehyde, and permeabilized with 0.1% Triton X-100. After blocking with 2% BSA for 45 min, coverslips were incubated overnight at 4°C with primary antibodies: anti-α-tubulin mouse monoclonal antibody (EMD Biosciences; San Diego, CA), and anti-β-tubulin rabbit monoclonal antibody (# 2128, Cell Signaling, San Francisco, CA). After washing, cells were incubated with Alexa Fluor 488- or Alexa Fluor 568-conjugated secondary antibodies (Invitrogen). Nuclei were visualized by inclusion of Draq5 (Thermo Scientific; Waltham, MA). Coverslips were mounted using Anti-Fade Fluoromount (Southern Biotech, Birmingham, AL), and the images were taken using an Olympus FluoView 1000 confocal microscope with 63 × 1.45 numerical aperture objective magnification and 565- and 488-nm laser wavelengths.

### Immunoblot analysis

Protein concentrations of cell lysates were determined using the DC Protein Assay (Bio-Rad; Hercules, CA). Equal amounts of protein (20 μg) from each cell lysate were resolved on Criterion TGX precast gels and transferred onto 0.45 μm nitrocellulose membranes (Bio-Rad). Membranes were blocked and incubated overnight at 4°C with primary antibodies: anti-total-β-tubulin (#2128; Cell Signaling), anti-β-I-tubulin (#T7816, Sigma), anti-β-III-tubulin (#MMS-435P, Covance), anti-β-IV-tubulin (#T7941, Sigma), anti-APC (#OP44, Calbiochem; San Diego, CA), anti-Rb (#9313, Cell Signaling), anti-pTyr204-ERK (#SC-7383, Santa Cruz Biotechnology; Santa Cruz, CA), anti-ERK1 (#SC-94, Santa Cruz), anti-Cyclin D1 (#2978, Cell Signaling), anti-Hsp27 (#2402, Cell Signaling), anti-pSer139-H2AX (#2577, Cell Signaling), anti-H2AX (#7631; Cell Signaling), anti-PARP (#9542, Cell Signaling), anti-ABCB1 (#517310, Calbiochem). After TBST washes (1X Tris-buffered saline, 0.1% Tween-20), membranes were incubated with corresponding horseradish peroxidase-conjugated secondary antibodies (Bio-Rad) for 1 h at room temperature. Proteins were detected by the enhanced chemiluminescence method (SuperSignal West Pico substrate; Pierce; Rockford, IL).

### Stability of tubulin protein

H630 cells were seeded in 6-well plates. Cells were treated with cycloheximide (10 mg/mL) and/or **1** (10 nM) for various times before being harvested for immunoblot analysis.

### Spheroid sprouting assay

A total of 500 HMEC-1 cells were suspended in culture medium containing 0.25% (w/v) carboxymethylcellulose and seeded in non-adherent round-bottom 96-well plates [43]. Cells were allowed to aggregate for 24 h at 37°C in 5% CO_2_. To perform the 3D collagen gel sprouting assay, type I collagen gel was made with 1 mg/mL collagen (Millipore), 0.25% (w/v) methylcellulose solution, 10% FBS and pH was adjusted to 7.4 with 5 M NaOH. Spheroids were collected and mixed with chilled type I collagen solution containing 2 nM **1**. Collagen containing approximately 6–10 spheroids was added into a pre-warmed 24-well plate and allowed to polymerize at 37°C for 1 h. MCDB-131 growth medium (200 μL) containing 2 nM **1** was pipetted on the top of gel and incubated at 37°C in 5% CO_2_. The number of sprouts per spheroid was counted manually under the microscope.

### Statistical analysis

All results are expressed as the mean ± S.D. and represent data from at least three independent experiments. Student's *t*-tests (two-tailed) were used to analyze differences between two groups, and *P* < 0.05 was considered statistically significant.

## SUPPLEMENTARY FIGURES


