# Supplementary material for: Bis-cyclopropane analog of disorazole C1 is a microtubule-destabilizing agent active in abcb1-overexpressing human colon cancer cells
Source: Oncotarget. 2015 Oct 19;6(38):40866–79. doi: 10.18632/oncotarget.5885 (PMC4747374; doi:10.18632/oncotarget.5885)
Supplement: Supplementary file 1 [file oncotarget-06-40866-s001.pdf]

SUPPLEMENTARY FIGURES

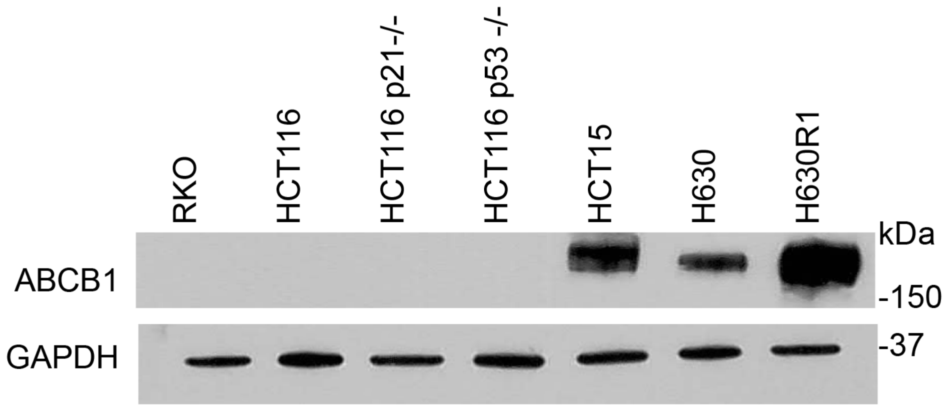

Supplementary Figure S1: Basal ABCB1 protein expression in human colorectal cancer cell lines.

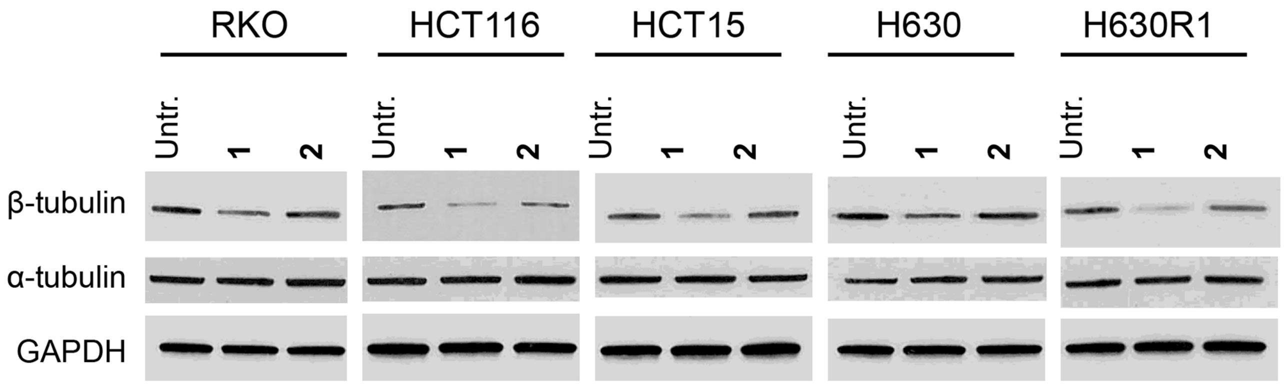

Supplementary Figure S2: Effect of 1 and 2 on β-tubulin protein levels. Cells were plated, treated for 24 h in the presence or absence of **1** and **2**, and harvested and processed for immunoblot analysis. A representative blot from four experiments is shown.
